# Alkali-Treated Alumina and Zirconia Powders Decorated with Hydroxyapatite for Prospective Biomedical Applications

**DOI:** 10.3390/ma15041390

**Published:** 2022-02-14

**Authors:** Damian S. Nakonieczny, Gražyna Simha Martynková, Marianna Hundáková, Gabriela Kratošová, Sylva Holešová, Jana Kupková, Lenka Pazourková, Justyna Majewska

**Affiliations:** 1 Nanotechnology Centre, CEET, VŠB-Technical University of Ostrava, 17. Listopadu 15, 708 33 Ostrava-Poruba, Czech Republic; grazyna.simha@vsb.cz (G.S.M.); marianna.hundakova@vsb.cz (M.H.); gabriela.kratosova@vsb.cz (G.K.); sylva.holesova@vsb.cz (S.H.); jana.kupkova@vsb.cz (J.K.); 2 Department of Biomedical Engineering, Silesian University of Technology, Akademicka 2A, 44-100 Gliwice, Poland; 3 IT4 Innovations, VŠB-Technical University of Ostrava, 17. Listopadu 15, 708 33 Ostrava-Poruba, Czech Republic; lenka.pazourkova@vsb.cz; 4 Department of Biosensors and Biomedical Signal Processing, Silesian University of Technology, Akademicka 2A, 44-100 Gliwice, Poland; justyna.majewska@polsl.pl

**Keywords:** zirconia, alumina, surface functionalization, alkaline etching, calcium deficient hydroxyapatite

## Abstract

The alumina and zirconia surfaces were pretreated with chemical etching using alkaline mixtures of ammonia, hydrogen peroxide and sodium hydroxide, and followed with application of the powder layer of Ca-deficient hydroxyapatite (CDH). The influence of etching bath conditions time and concentration on surface development, chemical composition and morphology of medicinal ceramic powders were studied. The following analyses were performed: morphology (scanning electron microscopy), phase composition (X-ray diffraction analysis), changes in binding interactions and chemical composition (FT-Infrared and Energy dispersive spectroscopies). Both types of etchants did not expose the original phase composition changes or newly created phases for both types of ceramics. Subsequent decoration of the surface with hydroxyapatite revealed differences in the morphological appearance of the layer on both ceramic surfaces. The treated zirconia surface accepted CDH as a flowing layer on the surface, while the alumina was decorated with individual CDH aggregates. The goal of this study was to focus further on the ceramic fillers for polymer-ceramic composites used as a biomaterial in dental prosthetics.

## 1. Introduction

Bioceramic materials, such as zirconia, alumina or groups of hydroxyapatites (HAp) are widely used in medical applications, especially in orthopedics and dental prosthetics [1,2,3]. The bioceramics used in medicine (e.g., for hips, knees and teeth replacements) may be nonporous and almost bioinert (e.g., zirconia or alumina), bioactive (e.g., dense HAp or glass–ceramic), resorbable (e.g., tricalcium phosphate) and porous for tissue ingrowth (e.g., porous Hap or HAp-coated porous metals) [4,5].

For medical applications, it is critical to obtain a correct connection between the bioceramics and the polymers or coated metals. Using alumina or zirconia ceramics as a component of the composites may be problematic due to the adhesion of its ceramic grains to the polymer resin. This is a consistent problem in cases, for example, in dental prosthetics and dentistry, where it is important to connect the patient’s enamel and prosthetic restoration with adequate abrasion [6,7]. Improvements to the adhesion are crucial for obtaining appropriate physicochemical properties, i.e., low abrasion, fatigue strength or resistance and thermal shocks [7,8]. For these reasons, surface modification to the zirconia and alumina remains a serious challenge to overcome. In recent years, several types of surface treatment—such as sandblasting, acid etching, sintering particles onto the surface, nanotechnological applications, laser irradiation, biofunctionalization and self-assembly—have been developed to produce a roughened ceramic surface that enables the fabricate of implants with good bioactivity and biocompatibility [9]. The modification of ceramics via simple chemical methods is commonly used to improve the adhesion to other materials but, in addition, may create new properties, such as biological or bacterio-fungicidal activity or enable its use as carriers for drugs such as chlorhexidine [10,11]. This is important because successful implants require not only the appropriate tissue compatibility, but also good antimicrobial properties that counter the formation of biofilms.

There is a significant amount of literature on the surface modification of biomaterials to improve their properties, such as the abrasion, biocompatibility and improvement of adhesion to human tissues, such as bones, and to enhance the construction of materials used for implants [12,13]. A reasonable quantity of research on the development of the surfaces of zirconia and alumina is available. There are two distinguishable groups of surface modifications: one based on physical methods, the other on chemical procedures. A frequently used chemical method is etching, especially acid etching, which can be performed by using hydrofluoric acid, nitric acid or sulfuric acid. An excellent benefit of using acid etching is that, regardless of the material’s shape and size, a homogenous roughening of the material can be obtained [14,15]. Chemical etching is the simplest and cheapest method for enabling the application of additional layers to the ceramic surface, i.e., oxides or nitride layers, and may allow for a controlled modification of the surface development; the application of additional functional groups, i.e., carboxyl, amine, or hydroxyl [16,17,18], is also possible.

Based on observations and accumulated data, chemical etching with alkaline compounds may be an appropriate method due to its low costs and the positive effects arising from developing the surface of the etched ceramics [19,20]. Studies by Riisager et al. determined information on the surface preparation of zeolites by etching with NaOH at different concentrations and processing times [19]. They found that, through the application of the NaOH, the microstructure can be affected; this includes an increase in the porosity of zeolites, a change in the crystalline characteristics and an improved suitability of the created active centers for catalytic applications that require increased surface development [19]. Moreover, Gutierrez et al. used a mixture of NaOH and Na_2_SiO_3_ for the alkaline preparation of concrete, ceramics and masonry waste for recycling and the preparation of a geopolymer for reuse in construction [20]. In their work, by using such an etching agent, they proved that it causes the fragmentation of post-industrial residues and, moreover, improves the mechanical performance in alkali-activated hybrid cements based on concrete, ceramics and masonry waste [20].

Improving the adhesion ability of ceramic grains can be achieved by an increase in the roughness of the ceramic surface, the formation of functional groups to ensure chemical connectivity or an oxidation [21,22,23]. Chemical processing is particularly popular for medical applications, such as applying layers of oxides, which are chemically bound to other materials [23,24]. In metal–ceramic composites, the greatest improvements in adhesion are obtained by oxidation [24]. In such a case, the strongest bonds are those in the oxygen bridges between the oxides in the alloys and the silicon atoms in the ceramics [23]. These bonds are due to the adherence of oxides, which develop on the surface of the alloys, within the initial firing cycle of the veneering process [23]. Various elements then diffuse across the contact zone between the alloy and the ceramic. A key factor in the polymer–ceramic composites is resin bonding via functional and mechanical groups. Zirconia and its adhesion to resins are particularly well studied [24]. Adhesion between zirconia and other ceramics or polymer–ceramics depends on several factors relating to the two different material phases. These include the chemical bonding, mechanical interlocking, wetting properties and the degree of interfacial stress generated by the thermal expansion mismatch and the glass transition temperature differences [25]. In the case of adhesion to ceramics, the surface is improved by a sandblasting process, while adhesion to polymers is obtained with better results when functional groups, e.g., hydroxyl moieties, are applied.

In recent years, calcium phosphates have been widely used as useful materials for biological and medicinal applications. Synthetic hydroxyapatite and its modifications can be used due to their similarity to and compatibility with human hard tissues (e.g., bones, enamel). Most research has mainly focused on the preparation of hydroxyapatite (HAp), but fewer studies have targeted calcium-deficient hydroxyapatite, Ca_10−x_(HPO_4_)_x_(PO_4_)_6−x_(OH)_2−x_ (0 < x < 1) (or CDH). This is a very promising material for biomedical utilization due to its resemblance to human hard tissue [26,27,28]. However, CDH exhibits a low mechanical strength, which limits its use mainly to low load-bearing applications, such as osteoconductive coatings on metallic or other ceramics prosthesis [29,30,31]. Phosphate ceramics exhibit considerably improved biological affinity and activity, but they contain poor crack growth resistance and poor mechanical properties (in terms of their strength and fracture toughness) in comparison to alumina and zirconia ceramics [29,30,31]. The chemical similarity of HAp to the inorganic components of human bones and teeth has led to extensive research in the field of hard tissue engineering [30]. According to earlier works, the use of CDH and HAp as cover layers on oxide ceramics can positively influence the osseointegration of biomaterials with bone and, thus, improve implant survival [31,32,33]. The group of Ca-deficient hydroxyapatite (CDH) is biocompatible and an inexpensive material, used for biomedical and non-biological applications. The decoration of clay surfaces with CDH can be prepared using precipitation methods under constant mixing or sonication conditions.

The main objective of this study is to prepare zirconia and alumina surfaces by alkaline etching to enable CDH decoration. The accumulated data on the surface preparation of aluminosilicates and oxide ceramics are analyzed; it is concluded that the proposed method—not yet used in medical applications for oxide ceramics—can achieve the desired result that improves the bonding to CDH. The treated ceramic, using different conditions, is first evaluated, and then calcium deficient hydroxyapatite is deposited onto the ceramics surface using precipitation techniques. The morphology, phase changes and bonding interaction are the key parameters that are evaluated, which lead to possible biomedical applications.

## 2. Materials and Methods

### 2.1. Sample Preparation

The powder samples were prepared from the ceramic powders: alumina (aluminum oxide, 99.9%, powder size 45 μm, 1344-28-11, Sigma Aldrich/Merck KGaA, Darmstadt, Germany) and zirconia (zirconium(IV) oxide, 99% purity excludes~2 HfO_2_, powder size 5 μm, CAS1314-23-4, Sigma Aldrich/Merck KGaA, Darmstadt, Germany). Samples were chemically etched in the following solutions without any additional treatment: a mixture of 35% ammonium hydroxide, NH_4_OH, with 35% hydrogen peroxide, H_2_O_2_ and a 8 and 6 M solution of sodium hydroxide, NaOH, mixed with 35% hydrogen peroxide, H_2_O_2_. All reagents were purchased from Avantor Chemicals, Radnor, PA, USA. The etching period was set at a 120 s duration in solution. The etching solution concentrations, ratios and etching times are given in Table 1 and Table 2.

**Figure 1:**
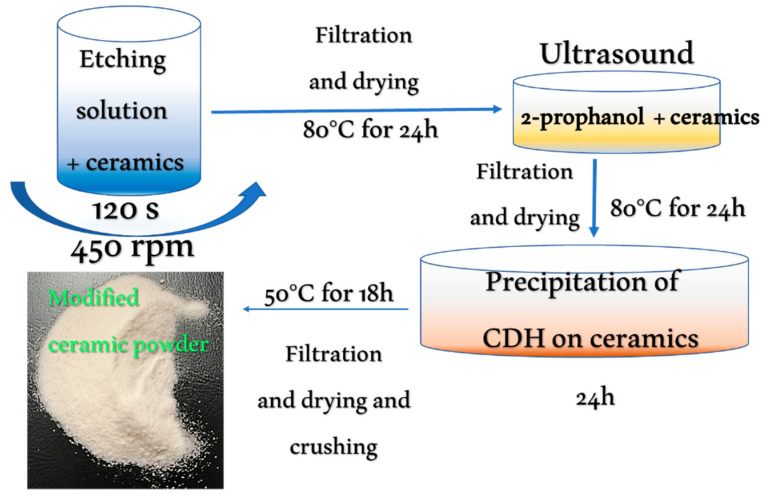
Schema depicting the preparation of the modified ceramics, including the preparation conditions.

The treatment procedure was conducted using the following steps, as represented by Figure 1. Ceramic powder samples were placed in a beaker with the etching solution and then mixed using a magnetic stirrer (450 rpm, at ambient temperature and atmospheric pressure). After the etching, the samples were quantitatively transferred to filter paper and washed with deionized water and then filtered under pressure. To remove the remaining etching solutions, each sample was filtered under pressure three times using a filter with a ceramic membrane and a paper filter (3 × 200 cm^3^ deionized water was used). The samples were then transferred to a dryer and dried, without a forced airflow, at 80 °C for 24 h. After cooling the samples to disintegrate any agglomerates, they were next added to a beaker without water and placed in an ultrasonic bath with 2-prophanol (Merck KGaA, Darmstadt, Germany) to homogenize them (using the parameters frequency = 37 kHz, power = 120%, time = 15 min and an ambient temperature, while a pulsation degassing program was employed).

Calcium chloride dihydrate (CaCl_2_·2H_2_O) and disodium hydrogen phosphate dodecahydrate (Na_2_HPO_4_·12 H_2_O) were both purchased from Lach-Ner, Co., (Neratovice, Czech Republic), and they were used as the precursors for preparation of the Ca-deficient hydroxyapatite (CDH) samples. Selected alkaline-modified samples of alumina and zirconia (both with mass 0.1 g) were mixed with a 500 mL solution of Na_2_HPO_4_·12 H_2_O (at concentration 7.2 mM). Next, 100 mL of CaCl_2_·2H_2_O (12 mM) solution was added dropwise for 1 h to the suspensions. After this time, the pH value was adjusted to 7.45 using 1 M HCl. The obtained precipitates were allowed to settle for 24 h. The solid products were then separated by filtration and dried overnight at 50 °C [4].

### 2.2. Materials Characterization

The prepared samples were tested in terms of their morphology, functional group characteristics and phase composition. The characterization of the morphology was performed using scanning electron microscopy (SEM). A high-resolution scanning electron microscope JEOL JSM-7610F + (JEOL, Akishima, Tokyo, Japan) with Schottky cathode and a microanalyzer Aztec Ultima Max 65 (Oxford Instruments, Abingdon, UK) were used to obtain these measurements. Both the secondary (SE) and backscattered electrons (BSE) were detected to enable the imaging and elemental mapping. Samples were prepared on stubs that were covered with carbon tape and coated with a conductive layer of gold to optimize the image resolution and avoid any possibility of charging.

The analysis of the functional group’s characteristics and the bonding interactions was performed using Fourier transform infrared (FTIR) spectroscopy. The FTIR spectra of all the samples were measured using the attenuated total reflectance (ATR) technique. The samples were pressed with a pressure device on the single-reflection diamond ATR crystal, and the spectra were collected using an FT-IR Nicolet iS50 (Thermo Scientific, Waltham, MA, USA) spectrometer with a Smart Orbit ATR accessory and a deuterated triglycine sulphate (DTGS) detector. The measurement parameters used were: spectral region = 4000–400 cm^−1^, spectral resolution = 4 cm^−1^, 64 scans, and Happ-Genzel apodization was used.

The X-ray powder diffraction analysis for the phase composition was performed using a RIGAKU Ultima IV (Rigaku, Tokyo, Japan) diffractometer with a scintillation detector, CuKα radiation source, NiKβ filter and a Bragg–Brentano arrangement. The samples were measured at ambient atmospheric pressure by use of the reflection mode (under the conditions 40 kV, 40 mA, 2°/min and 0.05 step). The database used to obtain the qualitative phase analysis was ICDD PDF-2/Release 2011 RDB.

## 3. Results

**Figure 2:**
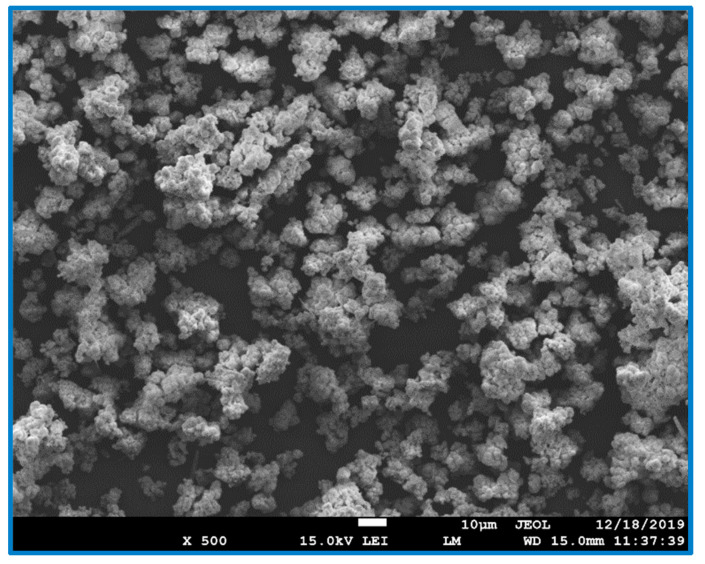
SEM image showing the morphology of the raw ZrO_2_ sample. The scale bar represents a length of 10 μm, and the magnification is 500×.

**Figure 3:**
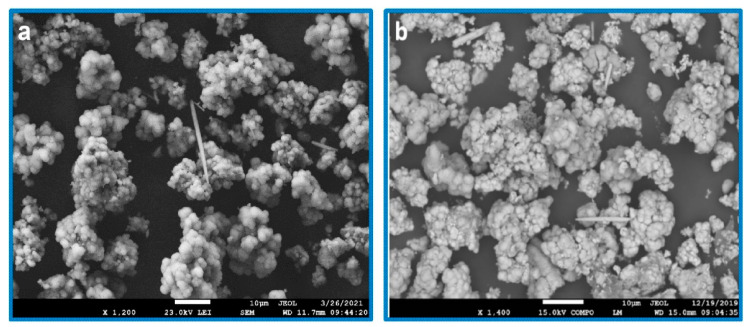
Comparison of the morphology of the etched ZrO_2_ samples using: (**a**) NH_4__ZrO_2__1 (magnification 1200×) and (**b**) 8Na_ZrO_2__1 (magnification 1400×), scale bar 10 μm.

The observations for the ceramic particle’s morphology, alongside the evaluation of the size and eventual agglomeration of the sample particles, was obtained using scanning electron microscopy. The observations of samples were divided into three parts: the original, the alkali-treated surface and the CDH deposited particles surface. The first sample analyzed was the original untreated zirconia sample, where smooth round particles with diameters around 1 μm were agglomerated into larger objects with 20–50 μm size (Figure 2). The modified samples were then analyzed, and the following treatment effects were observed for the zirconia samples. The samples etched in mixture of NH_4_OH:H_2_O_2_ are all agglomerated, and the degree of agglomeration decreases with an increasing etching time (Figure 3a). This is because the hydrogen peroxide has strong oxidizing properties, whereby the etching agent etches the crystal ceramics. In contrast, the samples etched in the mixture of NaOH:H_2_O_2_ are slightly different, (Figure 3b) since a higher number of agglomerated particles can be observed. Figure 3 depicts a comparison of the zirconia etched in a mixture of NH_4_OH:H_2_O_2_ and NaOH:H_2_O_2_, under the same conditions. Comparing the size and morphology of agglomerates, the particles are similar in nature.

**Figure 4:**
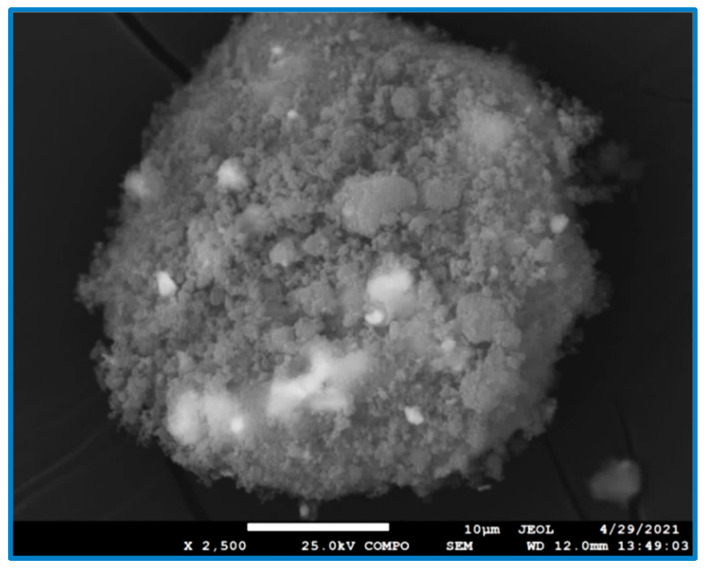
SEM image of ZrO_2_ without the etching, but doped with CDH (in BSE mode), at a magnification 2500×, scale bar 10 μm.

The next set of observed samples were treated zirconia ceramics with deposited CDH. In such a case, several phenomena were observed. For example, the original untreated zirconia was covered with a generous quantity of powdery CDH particles (Figure 4).

**Figure 5:**
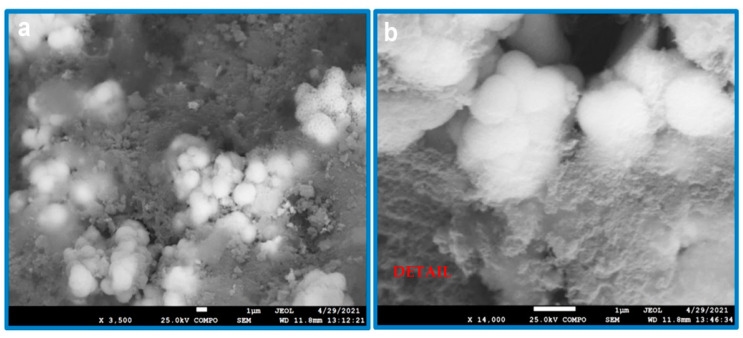
SEM images of the etched ZrO_2_ samples doped with CDH (sample CDH_NH_4__ZrO_2__1) with magnification: (**a**) 3500× and (**b**) detail at 14,000×, scale bar 1 μm.

Once the surface of the zirconia is treated, we can observe the different characteristics of the CDH deposition. All surfaces have approximately the same appearance (Figure 5): layer-like fine particles cover almost all the samples’ surface, which is a suitable condition for creating relatively continuous layers.

**Figure 6:**
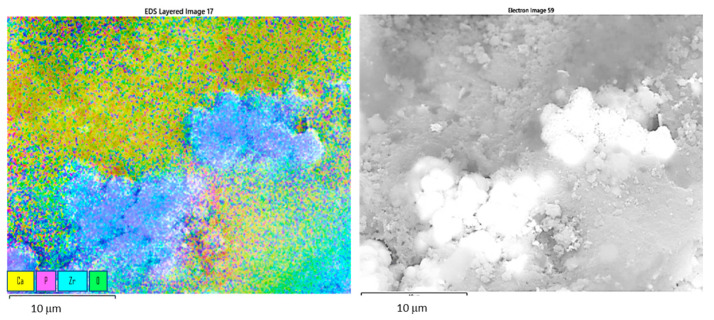
EDS mapping of the ZrO_2_ (CDH_NH_4__ZrO_2__1 sample) surface (**left**) and rough electron image of mapped area where the analysis took place (**right**): major elements on the surface etched and modified with CDH, Ca—yellow, P—magenta, Zr—cyan, O—green, scale bar 10 μm.

Elemental mappings of the surface (Figure 6) show the positions in which the CDH is deposited, or the uncovered zirconia particles are present.

**Figure 7:**
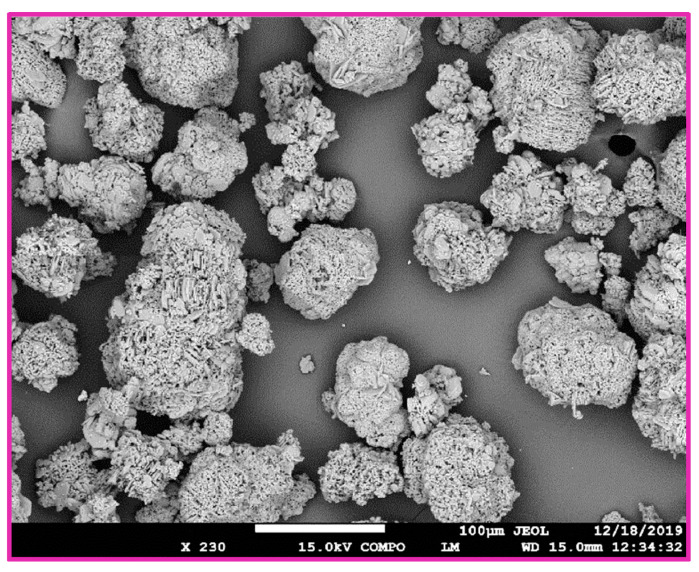
Morphology of the untreated Al_2_O_3_ sample with a magnification 230×.

**Figure 8:**
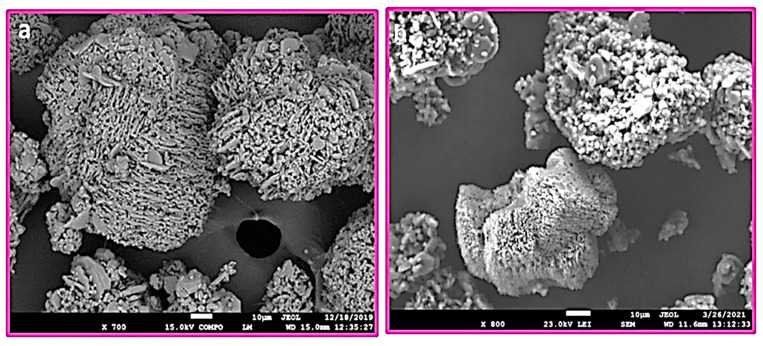
Comparison of the Al_2_O_3_ samples that are etched in a solution of: (**a**) NH_4_OH:H_2_O_2_ with magnification 700× (1:1, 120 s) NH4_Al_2_O_3__1 and (**b**) 8M NaOH:H_2_O_2_ with magnification 800× (1:1, 120 s) 8Na_Al_2_O_3__1.

Further investigations were performed on the aluminum ceramic samples (Figure 7). For the alumina samples, etching in a solution of NH_4_OH:H_2_O_2_ had a similar effect to the zirconia. The following observations were made for the alumina samples etched in NaOH:H_2_O_2_ mixtures compared to the case involving NH_4_OH:H_2_O_2_ solution: the surfaces are much less developed, which is especially evident, since the contours of whole alumina crystals remain without any decomposition (Figure 7), the degree of agglomeration is almost identical, and no other significant differences are apparent. Figure 8 shows the comparison of the alumina etched in NH_4_OH:H_2_O_2_ and in NaOH:H_2_O_2_.

**Figure 9:**
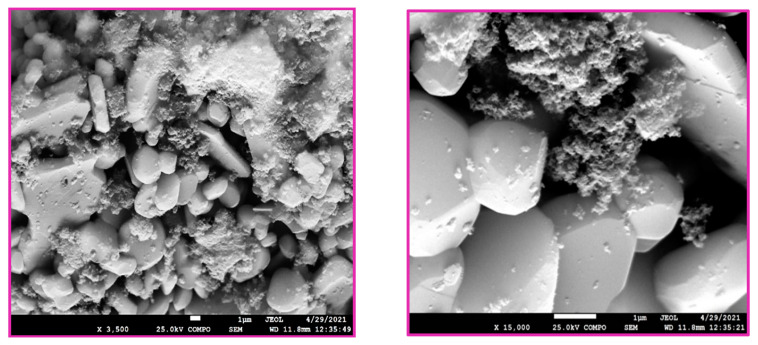
Comparison of the etched Al_2_O_3_ modified with CDH (CDH_NH_4__Al_2_O_3__1 sample) at a magnification: 3500× (**left**) and 15,000× (**right**).

**Figure 10:**
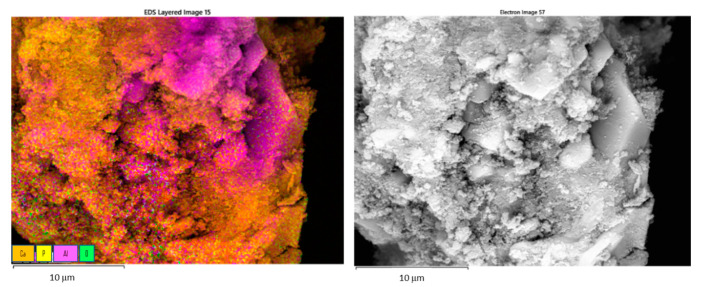
EDS mapping showing the major elements on the Al_2_O_3_ etched surface modified with CDH (**left**); and electron image of the scanned area where the analysis took the place (**right**); Ca—orange, P—yellow, Al—magenta, O—green, scale bar 10 μm.

Modification of the alumina surface with CDH creates a significant difference. The CDH crystals are positioned locally, and an extremely large area is not covered at all (Figure 9). This effect is identical for both etchants; however, the time of etching provides a more suitable condition for the growth of the CDH needle crystals. EDS mapping of the modified surface shows a large, exposed area of uncovered alumina (Figure 10). The CDH is variously located in grains or aggregates on (or around) the surface of the ceramic particles.

**Figure 11:**
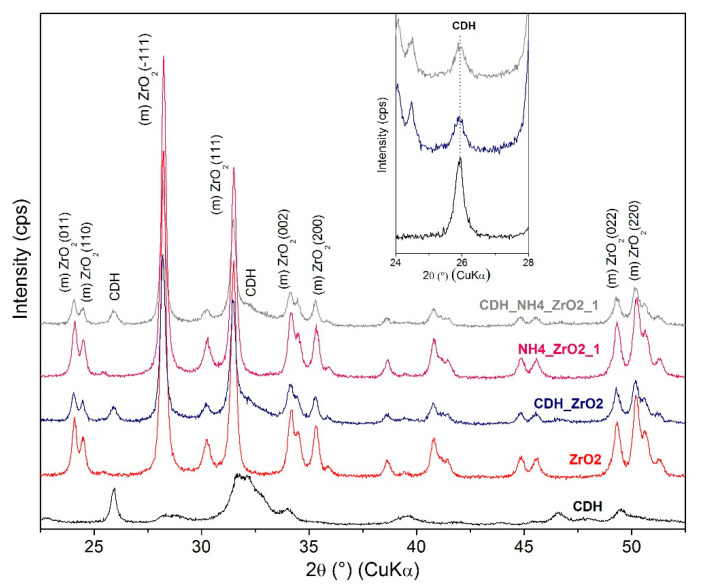
XRD patterns for the zirconia sample etched with NH_4_OH:H_2_O_2_ and denoted separate individual patterns that include pure CDH; the inset relates to the CDH 26° 2Θ peak.

**Figure 12:**
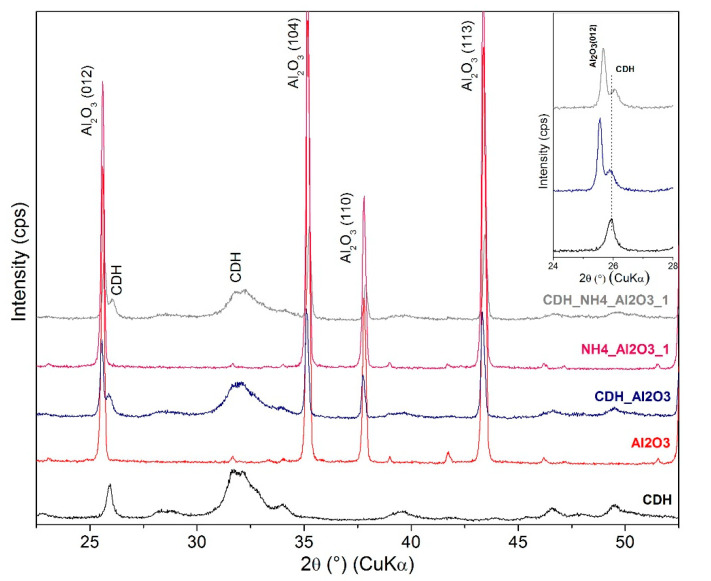
XRD patterns for alumina sample etched with NH_4_OH:H_2_O_2_ and denoted individual patterns and separate pure CDH and Al_2_O_3_ patterns and detail of CDH 26° 2Θ peak.

For a further study on the structure, an X-ray diffraction analysis was performed. This is based on the diffractograms obtained for the zirconia and alumina, in which no apparent effects from the etching process were found for the phase composition. For zirconia, the monoclinic ZrO_2_ (PDF Card No.: 01-083-0944) and tetragonal ZrO_2_ (t) (PDF Card No.: 01-080-0965) phases were identified (Figure 11). Additionally, minor diffraction peaks of HfO_2_ (PDF Card No.: 00-053-0560) were found that correspond with the quantity of oxide provided by the producer CAS 1314-23-4. In the case of alumina, both the original and all the treated samples show major and intensively pure α-Al_2_O_3_ phase peaks (PDF Card No.: 01-075-6776) (Figure 12) and the minor phases of the orthorhombic Al_2_O_3_ (o) (PDF Card No.: 01-076-8188) and AlO(OH) (PDF Card No.: 01-088-2351). All three phases appear as low-intensity, broad peaks that are generally considered as semicrystalline matter.

**Figure 13:**
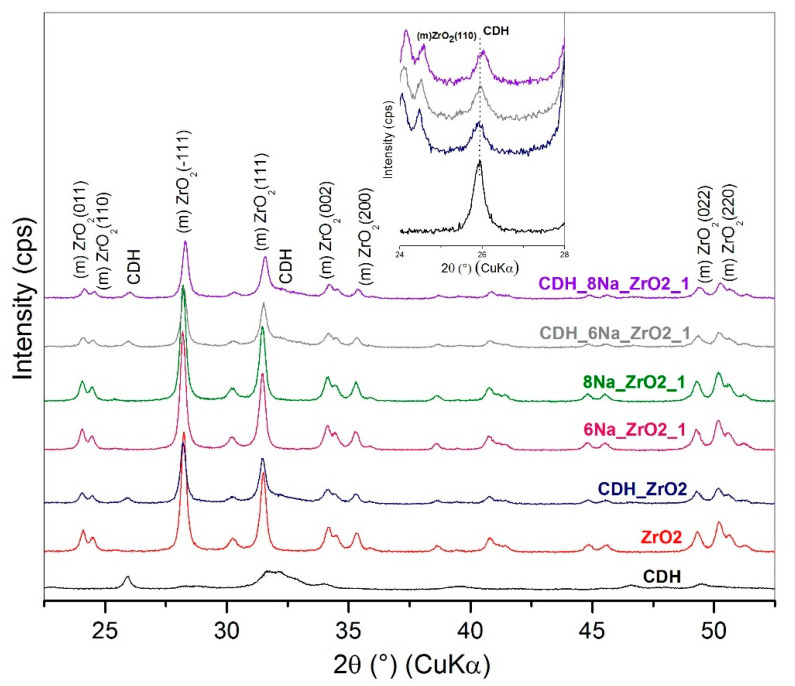
XRD patterns for zirconia sample etched with 6 M and 8 M of NaOH:H_2_O_2_ and denoted individual patterns and separate pure CDH and ZrO_2_ pattern and detail of CDH 26° 2Θ peak.

**Figure 14:**
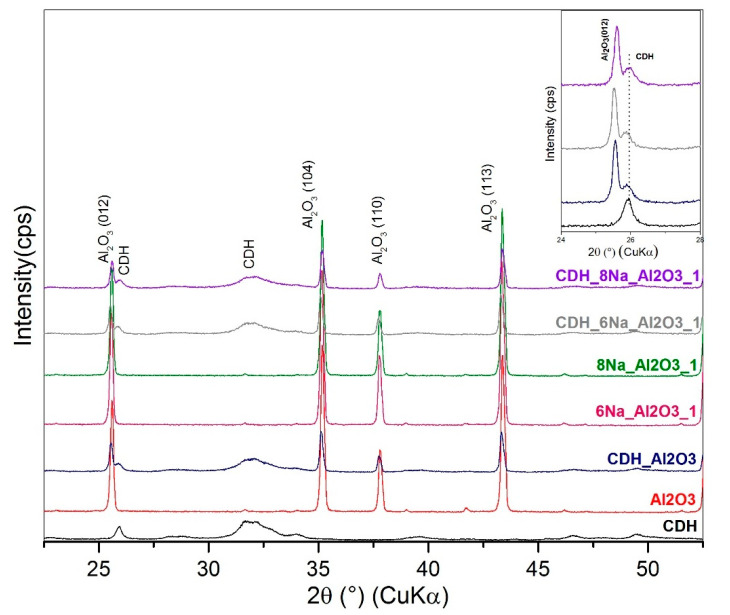
XRD patterns for zirconia sample etched with 6 M and 8 M of NaOH:H_2_O_2_ and denoted individual patterns and separate pure CDH and Al_2_O_3_ pattern and detail of CDH 26° 2Θ peak.

The Ca-deficient hydroxyapatite (CDH) phase was identified, and its characteristic diffraction (002) is prominent at approximately 26° 2Θ, as shown in Figure 11, Figure 12, Figure 13 and Figure 14. Evaluating the modified zirconia ceramics, the CDH peak is well visible, and it is not influenced by the other phases, and both etchants give similar results. In case of the alumina ceramics substrate, we observe small peaks of CDH that overlap with the Al_2_O_3_ (012) diffraction. On comparing the peaks with the ones observed for the zirconia ceramics, we can state that they are less intense and generally diffused.

Comparing the results from SEM and XRD analyses, we can state that XRD confirmed a fine CDH powder layer observed at SEM that is not well crystalline because the peaks of the Ca-deficient phase are broad and present low intensity, while the original ceramics are well defined in shapes in SEM images that corelate with XRD peaks of ceramics, that are narrow and intensive, and we can assume better crystalline state.

**Figure 15:**
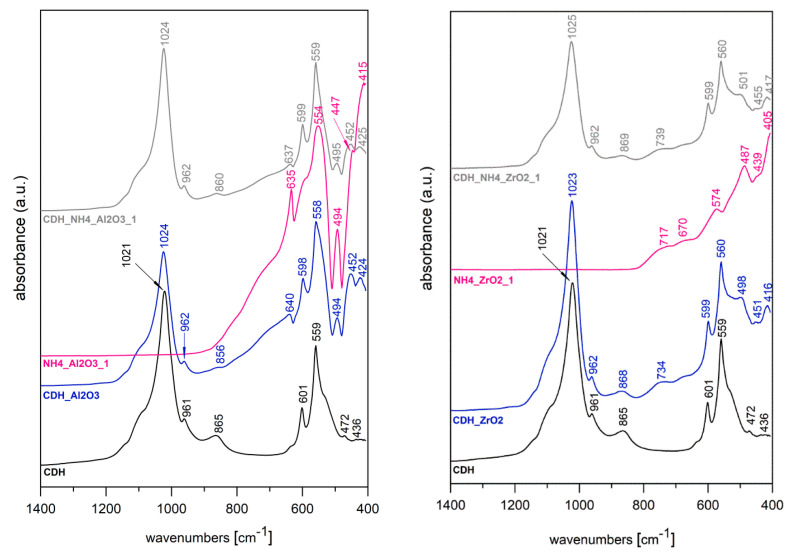
FTIR spectra for the modified powders samples: for the zirconia and alumina samples with and without CDH modification.

Figure 15 depicts the FTIR spectra for the eight samples. For the zirconia etched in a solution of NH_4_OH:H_2_O_2_ (1:1, 1:3), the following bands are identified: 717, 719, 670, 655, 574, 572, 487, 486, 439, 447 and 405 cm^−1^, which relate to the Zr–O bond vibrations in both zirconia samples [34,35,36,37,38,39]. In addition, the bands in the range 450–550 cm^−1^ correspond to the tetragonal zirconia [34,35,36,37,38]. In these spectra, additional bands relating to other functional groups are not seen. For the etched sample in a solution of NaOH:H_2_O_2_ (8 M NaOH, 6 M NaOH), the following bands are seen: 721, 721, 658, 658, 571, 573, 485, 486, 447 and 443 cm^−1^, which, as before, are associated with the vibration of the Zr–O bond. Again, no additional bands were found from other functional groups, e.g., from OH^-^. For alumina etched in a solution of NH_4_OH:H_2_O_2_ (1:1, 1:3), the following bands are discovered: 634, 636, 552, 557, 493, 495, 447 and 448 cm^−1^. These values correspond to the binding vibrations of Al-O, and the Al-O-Al in the vibrations of γ-Al_2_O_3_ and Al-OH [28,34]. Meanwhile, the characteristic absorption band at 413 and 416 cm^−1^ approximately relates to the range previously found elsewhere for Al-O, Al-O-Al and Al-OH [29,34]. For alumina modified with NaOH: H_2_O_2_ bath samples, the following bands occurred: 634, 635, 555, 554, 494, 494, 446, 447, 413 and 415 cm^−1^, which correspond to the same groups as alumina etched in NH_4_OH:H_2_O_2_ solution. An abundant amount of CDH is seen, as evidenced by the presence of the 868 cm^−1^ bands that are characteristic of CDH. We also observe band 1025 cm^−1^, which is for v^3^ PO_4_^3−^, and 962 cm^−1^ for v^1^ PO_4_^3−^. There remain two v^4^ PO_4_^3−^ that correspond to CDH 560 and 600 cm^−1^, but significant shifts of the CDH bands are not present in either case. For ZrO_2_, both the CDH and ZrO_2_ bands can be seen on the FTIR. The ZrO_2_ band is shifted from 486 cm^−1^ to around 501 cm^−1^, which is a relatively significant shift, but the CDH band does not move. The most intense Al_2_O_3_ bands are overlapped by the CDH, except for the band at 494 cm^−1^, but there is no shift. The last Al_2_O_3_ band shifts from 634 to 640 cm^−1^ but its intensity is quite reduced, which could also be due to the presence of the intense CDH bands.

The spectra of the samples treated with the NaOH etchant show very similar characteristics to those etched with NH_4_OH mixtures, therefore, they are not shown in this work.

The obtained results are part of a broader study on the modification of ceramic filler coatings dedicated to polymer–ceramic composites (PCC) and represent a continuation of our earlier work [40,41]. These fillers have potential applications as an input to PCCs and could be used in 3D printing for dentistry [41,42]. These types of materials may also have significant importance in the case of biomechanical mismatch in metallic alloys that are used for implants. Moreover, the prepared ceramic powders may find application in: (I) polymer–ceramic composites for implanted scaffolds in dental prosthetics, (II) carriers for active substances, i.e., gentamecin or chlorhexidine, which support implant penetration and protect against rejection by the organism and (III) barrier layers on the implant to prevent both bacterial and fungal infections [43,44,45].

## 4. Conclusions

In this paper, the study of strongly alkaline solutions in the treatment of medicinal ceramic powders, and the monitoring of changes in the phase composition and surface morphology, were performed. The two ceramic powders, alumina and zirconia, showed very slight changes when treated with the two etchant mixtures. However, the surface is prone to modifications via deposit layer of Ca-deficient hydroxyapatite.

In the case of zirconia, the morphology of the deposited layer is almost a continuous fine film that covers the ceramic surface relatively well. By evaluating the FTIR spectra, we observed that the ZrO_2_ band is shifted from 486 cm^−1^ to around 501 cm^−1^, which is a significant shift, but the CDH band does not shift. The interaction of CDH with the modified ZrO_2_ surface is thus evident; the alkaline treatment provides the zirconium surface with improved “wetting” properties.

In case of the alumina, the ceramic surface behaves more inertly, since the CDH modification is rather local on the edges and does not remain on the major surface. The CDH powder is positioned locally, and a very large area is not covered at all.

In conclusion, a favorable alkali-treated surface, which is determined to be zirconia, may act as an excellent candidate for applications in medicine as bioceramics used in prosthetics.

## Figures and Tables

**Table 1:** Experimental conditions of the alkaline-treated zirconia ceramic samples and their CDH modification.

Etching Agent	Reagent’s Volume Ratio	Name of the Samples	
		ZrO_2_	ZrO_2_ + CDH
35% NH_4_OH 35% H_2_O_2_	1:1	NH_4__ZrO_2__1	CDH_NH_4__ZrO_2__1
	1:3	NH_4__ZrO_2__0.3	CDH_NH_4__ZrO_2__0.3
8 M NaOH 35% H_2_O_2_	1:1	8Na_ZrO_2__1	CDH_8Na_ZrO_2__1
6 M NaOH 35% H_2_O_2_	1:1	6Na_ZrO_2__1	CDH_6Na_ZrO_2__1

Note: The sample name creation, e.g., 6Na_ZrO_2__1 is etchant _ base ceramic _ concentration or, e.g., CDH_6Na_ZrO_2__1 is Ca-deficient hydroxyapatite _ etchant _ base ceramics _ concentration.

**Table 2:** Experimental conditions of the alkaline-treated alumina ceramic samples and their CDH modification.

Etching Agent	Reagent’s Volume Ratio	Name of the Samples	
		Al_2_O_3_	Al_2_O_3_ + CDH
35% NH_4_OH 35% H_2_O_2_	1:1	NH_4__Al_2_O_3__1	CDH_NH_4__Al_2_O_3__1
	1:3	NH_4__Al_2_O_3__0.3	CDH_NH_4__Al_2_O_3__0.3
8 M NaOH 35% H_2_O_2_	1:1	8Na_Al_2_O_3__1	CDH_8Na_Al_2_O_3__1
6 M NaOH 35% H_2_O_2_	1:1	6Na_Al_2_O_3__1	CDH_6Na_Al_2_O_3__1

## Data Availability

Not applicable.
